# Combined Cryptococcal and Bacterial Meningitis in an Immunocompetent 15‐Year‐Old: A Case Report

**DOI:** 10.1155/crdi/4601111

**Published:** 2026-05-24

**Authors:** Martha Ann Mbonye, Edna Joyce Nagaddya

**Affiliations:** ^1^ Internal Medicine Department, Kagando Hospital, Kasese, Uganda

**Keywords:** bacterial meningitis, coinfection, cryptococcal meningitis, immunocompetent host, low-resource settings, neurology, tropical medicine

## Abstract

**Introduction:**

Cryptococcal meningitis is a serious fungal infection of the meninges, typically seen in immunosuppressed patients. The occurrence of cryptococcal meningitis in immunocompetent individuals is very rare. Coinfection with bacterial meningitis is even more unusual.

**Primary Diagnoses, Interventions, and Outcomes:**

We report the case of a 15‐year‐old male who presented to a district hospital in rural Western Uganda with a 2‐week history of severe headache, altered mentation, vomiting, and high‐grade fever unresponsive to initial empirical treatment for cryptococcal meningitis. Subsequent evaluation revealed a combined fungal and bacterial meningeal infection. Tailored antifungal and antibacterial therapy led to significant clinical improvement, though residual neurological sequelae persisted at discharge.

**Conclusion:**

This case highlights the diagnostic and therapeutic challenges in low‐resource settings. High clinical vigilance, even for those with no comorbidities or immunosuppression, and the use of easily available diagnostic modalities can help with early diagnosis and better outcomes.

## 1. Introduction

An encapsulated saprophytic yeast called *Cryptococcus* is the source of cryptococcosis, an uncommon illness that is often seen in immunosuppressed people. The ability of this yeast to spread widely and infect the majority of organs in patients with severe immunosuppression has been emphasized. Clinical disease is seen commonly in the central nervous system (CNS) and lungs, and to a lesser extent, the skin, prostate, eyes, and joints.

Cryptococcal meningitis (CM) is a fungal infection of the tissues covering the brain and spinal cord called meninges. CM predominantly affects individuals with impaired cell‐mediated immunity, particularly those living with human immunodeficiency virus/acquired immunodeficiency syndrome (HIV/AIDS). In such populations, it remains a leading cause of meningitis‐related deaths, especially in sub‐Saharan Africa.

Its occurrence in immunocompetent patients is rare, and a combined infection with bacterial meningitis is even rarer. With only a few cases described in the literature, concurrent cryptococcal and bacterial meningitis remains an uncommon clinical entity that requires a high index of suspicion and comprehensive evaluation.

Due to its indolent course and nonspecific early symptoms, delays in recognition and treatment are frequently encountered in resource‐limited settings, often with significant neurological complications and poor outcomes.

## 2. Case Presentation

From the Department of Internal Medicine:

### 2.1. Patient Information

A 15‐year‐old previously healthy male from Uganda presented initially to St. Paul’s Health Centre IV with a 1‐week history of severe headache and 2‐day history of photophobia and altered mentation. There was no prior head trauma, chronic illnesses, or administration of any long‐term medications. There was no prior exposure to steroids or other immunosuppressive medications. There were no known drug allergies. He had had no sexual partners in his life. He did not drink alcohol, smoke tobacco, or use illicit drugs. There was no significant family history of neurological, rheumatologic, cardiovascular, or renal disease. The patient lived and received his medical care in a rural setting. His family were crop and poultry farmers.

### 2.2. Clinical Findings

Complete blood count showed mild leukocytosis and thrombocytopenia; malaria blood smear and rapid diagnostic testing were negative; HIV rapid test was negative. Initial lumbar puncture was performed at the referring facility, but cerebrospinal fluid (CSF) analysis was not complete. CSF and serum cryptococcal antigen tests were both positive.

The patient was empirically started on amphotericin B and fluconazole for presumed CM but showed no clinical improvement. Following a 1‐week admission, the patient was referred to Kagando General Hospital for renal function monitoring and further evaluation.

On arrival at Kagando General Hospital, he had developed projectile vomiting, lethargy, and persistent high‐grade fevers. His family reported uncoordinated speech and abnormal eye movements. A review of systems revealed a productive cough and neck pain radiating to the lower back.

The temperature was 38.6°C, the heart rate was 126 beats per minute, the blood pressure was 145/94 mmHg, the respiratory rate was 22 breaths per minute, and the oxygen saturation was 96% on room air. He was severely dehydrated.

The patient was lethargic but arousable; he was variably oriented to self but not to situation, location, or time. His Glasgow Coma Scale score was 10 out of 15: eye opening was spontaneous, but he did not follow commands or reliably withdraw in response to pain and had decorticate posturing. His pupils were dilated, unequal, and sluggishly reactive to light. Nystagmus and left esotropia were also noted. He had hyperreflexia and hypotonia in all of his limbs. There was no gaze preference or hemineglect.

There was no evidence of jugular venous distention or edema. There were no rales, and the abdominal examination was normal.

### 2.3. Diagnostic Assessment

The following repeat investigations were done on arrival.

The blood smear showed no malaria parasites, and the rapid test for malaria was negative as well. The RBS was normal.

The laboratory urinalysis was normal. The urine TB LAM was negative.

*Salmonella* IgG and IgM were positive. The stool analysis revealed no ova or cysts.

The repeat serum cryptococcal antigen test result was positive. Quantitative titers were not available due to laboratory limitations. *Cryptococcus* species identification and fungal culture speciation were not available at the treating facility.

The HIV serology results were negative with a normal CD4 count of 864 cells//μL. The results of the syphilis rapid plasma reagin test were negative.

Other laboratory test results are shown in Table 1.

**TABLE 1:** Laboratory data.

Variable	Reference range, this hospital	On current admission, this hospital
White cell count (× 10^9^ per liter)	5–19	13.71
Differential count (× 10^9^ per liter)		
Neutrophils	1.0–8.5	12.82
Lymphocytes	2.3–14.4	0.76
Monocytes	0.0–0.95	0.12
Hemoglobin (g/dL)	9.5–14.1	14.7
Hematocrit (%)	30.0–40.0	39.19
Platelet count (× 10^9^ per liter)	150–450	260
Potassium (mmol per liter)	3.5–5.5	2.7
Sodium (mmol per liter)	135–145	129.1
Chloride (mmol per liter)	98–108	105.1
Calcium (mmol per liter)	2.1–2.5	13.8

### 2.4. Therapeutic Interventions and Outcome

Intravenous high‐dose ceftriaxone 2 g OD was started to treat the salmonellosis and possible bacterial meningitis, pending CSF analysis.

He was also started on high‐dose fluconazole 600 mg OD to cover the cryptococcal infection, intravenous paracetamol 1 g TDS to act as antipyretic, and 40 mEq of 10% potassium chloride solution slow infusion to correct the hypokalemia.

The patient, however, developed a productive cough and continued spiking very high fevers.

There was an acute episode of tonic seizures lasting 1 min, involving both upper limbs. The patient was administered a stat dose of intravenous diazepam 10 mg that stopped the convulsion. Intravenous mannitol 10 g TDS was administered empirically to lower intracerebral pressure based on clinical presentation (worsening headache, projectile vomiting, seizures, and altered level of consciousness).

The lumbar puncture was repeated. Opening pressure could not be measured due to a lack of a manometer at the facility. Cerebrospinal analysis showed the following:

 Macroscopically, turbid
 Microscopically, 17–20 pus cells
  2‐3 yeast cells
 Total protein: 1.2 g/dL (reference range: 0.15–0.40 g/dL)
 Glucose: 37.8 mg/dL (reference range: 45–72 mg/dL)
 White cell count: 50 × 10^6^/L (reference range: less than 5 × 10^6^/L)
  Granulocytes: 55%
  Lymphocytes: 45%
 CSF CrAg positive, titers not available
 CSF India ink positive
 CSF Gram stain showed Gram‐positive rods and Gram‐negative rods
 Ziehl–Neelsen stain: No AFBs

The findings supported a diagnosis of concurrent cryptococcal and bacterial meningitis.

Intravenous amphotericin B 50 mg OD (1 mg/kg/day) for 7 days and Epicephin 2 g BD for 14 days were then initiated. Oral fluconazole dose was then increased to 600 mg BD (24 mg/kg/day). Intravenous paracetamol 1 g TDS was maintained.

In view of the new cough, intravenous metronidazole 500 mg TDS and ampicillin 1 g QID were initiated to manage aspiration pneumonia.

Neuroimaging could not be performed (no in‐hospital CT scan and patient not yet stable enough to be transferred).

Serial RBS values were always within normal range; type 2 DM was not a concern. There were no new seizures, so no therapeutic LPs were performed. Serum electrolytes were repeated and showed a hypokalemia of 2.13 mmol per liter. KCl infusions were continued intermittently.

Physiotherapy exercises were started to encourage ambulation. He was still weak and unable to sit unsupported; the exam revealed left hemiparesis. There was also suspicion of right‐sided hearing loss and bilateral visual loss based on an inability to identify objects or people right in front of him.

The patient survived his 3‐week admission. Over the course of his admission, he had suffered anorexia and weight loss, generalized weakness, dizziness, right upper quadrant pain, loose stools, and dark urine. At discharge, he was oriented and able to sit up in bed and converse. However, the left hemiparesis and vision and hearing impairment persisted. He returned home on oral fluconazole 400 mg BD for the next 8 weeks.

Audiometry, fundoscopy, and perimetry were scheduled to be done as an outpatient. The patient was unfortunately lost to follow‐up after discharge.

A detailed timeline of clinical events, diagnostic procedures, therapeutic interventions, and outcomes is summarized in Table 2.

**TABLE 2:** Timeline of clinical course.

Day 1	Onset of headache

Days 2–7	Progressive photophobia and altered mentation

Day 8	Presentation to St. Paul’s Health Centre IV Lumbar puncture done, CSF not analyzed Cerebrospinal fluid and serum CrAg positive Empirical amphotericin B and fluconazole initiated

Day 15	Worsening headache, projectile vomiting, fevers, altered mental status, and progressive lethargy. Referral to Kagando General Hospital for further evaluation Repeat serum cryptococcal antigen test result positive Intravenous high‐dose ceftriaxone, fluconazole, paracetamol, and potassium chloride solution initiated

Day 16	The patient developed a productive cough and continued spiking very high fevers. There was an acute episode of tonic seizures lasting 1 min, involving both upper limbs. The lumbar puncture was repeated.

Day 17	A diagnosis of concurrent cryptococcal and bacterial meningitis was made.

Day 19	Paracetamol stopped (the headache and fevers had subsided) No new seizures. No therapeutic LPs performed

Day 25	Serum electrolytes were repeated and showed a hypokalemia of 2.13 mmol per liter. KCl Infusions were continued as before.

Day 28	The patient was oriented and able to converse. Left hemiparesis noted. Right‐sided hearing loss and bilateral visual loss suspected. The antibiotic course was completed.

Day 36	Discharged

#### 2.4.1. Differential Diagnosis

This patient had a complex neurologic syndrome that developed over a 3‐week period. The initial presentation was presumed CM based on the profound neurologic manifestations and the positive serum cryptococcal antigen test. Hypoglycemia and cerebral malaria were ruled out.

The working differential diagnoses were as follows:

● Acute bacterial meningitis
● CM
● Septicemia with fungemia

#### 2.4.2. Final Diagnosis

The final diagnosis was combined cryptococcal and bacterial meningitis in an immunocompetent patient (first presentation).

## 3. Discussion

CM is the most common form of adult meningitis in regions with a high prevalence of HIV infection. The global incidence rate is 278,000 cases of cryptococcosis and 223,100 cases of CM [1]. Globally, CM claims 181,100 lives per year, with sub‐Saharan Africa accounting for 75% of these deaths, and is responsible for 15% of AIDS‐related deaths [1].

CM is classically associated with advanced HIV disease and other states of defective cell‐mediated immunity [2]. Its occurrence in individuals without known immunosuppression is uncommon [3], and dual infection with bacterial meningitis is even rarer. Reports of simultaneous bacterial meningitis and CM are scarce in the literature and are usually described in severely immunocompromised hosts. The recognition of this combination in an immunocompetent patient is therefore unusual and clinically instructive.

### 3.1. Rare Presentation of CM

CM resulting from hematogenous dissemination of *Cryptococcus neoformans* is more commonly observed in immunocompromised patients. In immunocompetent patients, *Cryptococcus gattii* is thought to have a greater predilection than *Cryptococcus neoformans* [4]. However, an Indiana University study found that the majority of immunocompetent patients both in retrospective hospital cases and PubMed searches had *Cryptococcus neoformans* [5]. A prospective descriptive study of CM in HIV‐uninfected patients in Vietnam without underlying comorbidities showed that most patients had *C. neoformans* [6]. Similarly, a study of 200 HIV‐infected patients with CM in Kampala, Uganda, found 0% prevalence of *C. gattii* [7]. Therefore, based on geography, exposure, and host immune status, *C. neoformans* is the most likely culprit in this case.

Cases in immunocompetent individuals, although rare, are increasingly being reported, highlighting the pathogenic potential of this fungus even in the absence of overt immunosuppression. Increased environmental exposure and genetic host susceptibilities have been put forward as emerging contributors. Infection is via inhalation from the environment, that is, high‐level exposure to soil contaminated with avian excreta [8, 9]. Farming activity was a real risk factor in this patient. Although *C. neoformans* enters the body through the lungs, the CNS is the main site of evident clinical infection [9].

The occurrence of CM in a previously healthy patient can also be assigned to rare primary immune defects or uncommon autoimmune diseases such as idiopathic CD4+ lymphopenia (in 27% of cases), alveolar proteinosis, monogenic disorders (GATA2 mutations), and polygenic modifiers (Fcg receptor II polymorphism) [9, 10]. An association with anti‐granulocyte‐macrophage colony‐stimulating factor auto‐antibodies and idiopathic CD4 lymphocytopenia has also been put forward [11].

### 3.2. Coinfection

Dual bacterial and cryptococcal meningeal infections are exceedingly rare. The first case of dual infection in the English literature was described with *Cryptococcus neoformans* and *Streptococcus pneumoniae* in the CSF of an AIDS patient in 1998. The finding of pneumococci, in the absence of inflammatory cells in CSF, suggests the terminal event was fulminant pneumococcal meningitis in the setting of chronic CM [12].

Our case illustrates a rare presentation of CM in an immunocompetent host, further complicated by bacterial coinfection. The initial CSF analysis revealed cryptococcal antigen positivity. The subsequent identification of Gram‐positive rods and Gram‐negative rods in the CSF confirmed the bacterial meningitis.

The temporal relationship between cryptococcal and bacterial meningitis in this case remains uncertain. Early phase CM often presents with nonspecific symptoms and minimal CSF inflammation, so diagnostic delay has been assumed. Bacterial meningitis could have been present earlier but not detected due to an incomplete initial CSF analysis, and thus treatment was delayed. Alternatively, a secondary or nosocomial bacterial meningitis could have developed later, facilitated by immune dysregulation from cryptococcosis or been introduced via the first lumbar puncture done at the referring facility.

The immune dysregulation and evasion strategies employed by *Cryptococcus neoformans* could have facilitated bacterial invasion of the meninges. *Cryptococcus* avoids host macrophages by using multiple virulence factors such as a polysaccharide capsule, melanin, secreted enzymes (urease and phospholipases), and phenotypic plasticity in order to cross the blood–brain barrier and grow in a nutrient‐poor environment [13]. The breach in the blood–brain barrier creates a favorable milieu for polymicrobial infection and proliferation. The subsequent bacterial superinfection offers an explanation for the unusual clinical course.

This case supports the importance of performing both the Gram‐ and India‐ink stains and agar cultures on CSF specimens, even when biochemical and cellular parameters are normal. The overlap of symptoms such as fever, headache, and altered mentation complicates timely diagnosis.

### 3.3. Polymicrobial Gram‐Positive and Gram‐Negative Bacterial Meningitis

Salmonellosis, caused by a Gram‐negative bacillus of the Enterobacteriaceae family, typically presents with gastroenteritis. It is possible for *Salmonella* to break through the abdominal barrier, enter the bloodstream, invade the meninges, and persist in the subarachnoid space, using virulence factors such as intracellular invasion, polysaccharide capsule, and fimbriae, resulting in CNS infections such as meningitis, subdural effusion, empyema, or brain abscess [14]. Patients usually present with headache, fever, altered sensorium, and stiff neck. While *Salmonella* meningitis is rare, suspicion is high in this patient due to the high endemicity of typhoidal strains in sub‐Saharan Africa. This clinical picture is further reinforced by the presence of Gram‐negative rods in the CSF and the patient’s rapid neurological deterioration, both of which are characteristic of this aggressive infection.

The possibility of *Salmonella* meningitis did not change the management; a broad‐spectrum third‐generation cephalosporin was initiated from the receiving facility in view of the positive *Salmonella* IgG, and the dose was adjusted on confirmation of bacterial meningitis.

*Listeria monocytogenes* is the primary Gram‐positive rod responsible for causing meningitis in adults with compromised immune systems, the elderly, and sometimes young adults with weakened cell‐mediated immunity. It is notably resistant to cephalosporins and requires treatment with ampicillin, which had been started for aspiration pneumonia [15].

The clinical improvement seen in this patient following initiation of high‐dose ceftriaxone and ampicillin could support a degree of suspicion of *Salmonella* and *Listeria* meningitis.

### 3.4. Presentation

Subacute meningoencephalitis is how CM often manifests. At the first presentation, the clinical symptoms may be few or vague. In cases of HIV, the interval between the onset of symptoms and their presentation is usually between one and two weeks, but in cases of non‐HIV, it is between six and twelve weeks.

Due to the significant inflammatory burden, in part attributed to the polymicrobial bacterial meningitis, this patient presented with the typical neurological symptoms such as bilateral headaches and altered mental status along with lethargy, fever, stiff neck, nausea, and vomiting.

Diplopia and photophobia are two early signs of the illness seen in this patient, while subsequent signs included reduced visual acuity (induced by high CSF pressure or compression of the optic nerve and tracts) [16], ataxia, aphasia, chorea, tinnitus, and phonophobia. Anorexia and weight loss, generalized weakness, dizziness, right upper quadrant pain, soft stools, and dark urine were further outcomes. The disease progressed to confusion, generalized seizures, and reduced level of consciousness.

### 3.5. Diagnosis

At the referring health facility, only the serum and CSF cryptococcal antigen tests were done, so empiric treatment with amphotericin B and fluconazole was initiated. Following referral to the tertiary hospital, the patient presented with worsening symptoms: projectile vomiting, high‐grade fever, tonic seizures, and progressive lethargy. Initial assessment revealed severe dehydration, uncoordinated speech, abnormal eye movements, and a Glasgow Coma Scale score of 10. Complete blood count, RBS, and malaria blood smear were done as initial assessment. A lumbar puncture was done, and a CSF analysis revealed a mixed meningeal infection: positive India ink stain for *Cryptococcus*, Gram‐positive and Gram‐negative rods, elevated protein, and hypoglycorrhachia.

The diagnosis in this patient relied heavily on basic but high‐yield investigations. India ink staining and cryptococcal antigen testing of CSF established the diagnosis of CM despite the absence of classical risk factors. The clinical deterioration prompted reconsideration of the initial diagnosis and subsequent identification of Gram‐positive and Gram‐negative organisms on CSF Gram stain. With just this preliminary knowledge, even with no bacterial culture and speciation, targeted therapy could be initiated appropriately.

This case shows that there is a real possibility of indolent CM complicated by acute bacterial coinfection in immunocompetent individuals. When clinical trajectory deviates from expected recovery, there is need for a broad diagnostic approach, particularly in low‐resource settings. Diligent clinical assessment and empiric treatment tailored to cover both infections can significantly impact outcomes.

### 3.6. Management

An integrated approach is required to manage coinfections. Antifungal therapy with amphotericin B and fluconazole or flucytosine remains the cornerstone of CM treatment [17, 18]. The ASM/ECMM/ISHAM Global Guideline for the Diagnosis and Management of Cryptococcosis (2024) recommends that HIV‐associated CM be treated with liposomal amphotericin B 10 mg/kg as a single dose, with 14 days of flucytosine 25 mg/kg four times a day and fluconazole 1200 mg daily in low‐income settings; this induction therapy has however not been trialed in non‐HIV‐associated CM or other non‐CNS cryptococcosis syndromes [19]. The Advancing Cryptococcal Meningitis Treatment for Africa (ACTA) trial concluded that 1 week of amphotericin B plus flucytosine and 2 weeks of fluconazole plus flucytosine were effective as induction therapy for CM in resource‐limited settings [18].

High‐dose ceftriaxone, an empiric broad‐spectrum antibiotic, is essential to address the bacterial meningitis. Bactericidal antibiotics for a minimum of 3 weeks should effectively clear Gram‐negative bacillary meningitis.

In line with current guidelines, this patient received intravenous amphotericin B, high‐dose fluconazole [20], and ceftriaxone to target both cryptococcal and bacterial pathogens. Adjunct therapies included mannitol to lower intracranial pressure, KCl infusions to counter hypokalemia, and metronidazole to address the aspiration pneumonia.

### 3.7. Outcome

CM in young, previously healthy patients is likely under‐recognized. Determining risk factors for cryptococcosis in these patients remains elusive. This patient’s farming exposure and likely high‐level exposure to soil contaminated with avian excreta provide a plausible environmental source of infection. Despite confirmed HIV negativity and normal CD4 count, the possibility of an unidentified immune defect could not be excluded, as testing was not available.

Immunocompetent individuals with CM often present with indolent neurological disease. Patients with CM who do not exhibit the typical meningitis symptoms and who do not have the major risk factor (immunosuppression) often have poor outcomes because delayed diagnosis and misdiagnosis are common, and patients may suffer from serious complications with high mortality [21].

So, *Cryptococcus* should always be kept as a possible cause of meningitis particularly in the developing world even if there are no comorbidities or immunosuppression. Given how catastrophic bacterial meningitis can be, there is a strong argument for early empiric antibacterial coverage, especially in settings where CSF analysis could be delayed and monitoring limited.

Despite treatment, the patient suffered left hemiparesis, suspected right‐sided hearing loss, and bilateral visual impairment. Balance and psychiatric issues were not exclusively ruled out.

### 3.8. Diagnostic and Therapeutic Challenges

We present a rare case of concurrent cryptococcal and bacterial meningitis in an immunocompetent adolescent, highlighting diagnostic and therapeutic challenges in resource‐limited settings where advanced diagnostic modalities are unavailable.

This patient had a low GCS at presentation, new focal neurologic deficits, and delayed initiation of treatment. A better prediction of the prognosis of this patient could have been made if CSF and serum cryptococcal antigen titers or opening pressures upon lumbar puncture were measured.

The initial diagnosis of CM was based on positive serum and CSF cryptococcal antigen tests which are inexpensive and widely recommended in endemic regions [22]. The bacterial coinfection was detected only after a lumbar puncture was repeated and comprehensive diagnostic evaluations using Gram, India ink, and Erlich–Ziehl–Neelsen stains followed. The limited availability of CSF and blood cultures and sensitivity, fungal speciation, and other rapid immunodiagnostic assays contributed to diagnostic and treatment delays. HIV and CD4 testing confirmed immunocompetence in this case. Further immunologic evaluation, including testing for anti‐GM‐CSF antibodies or idiopathic CD4 lymphopenia, was not available.

Brain imaging could not be obtained due to lack of CT/MRI availability at the facility and the patient’s initial instability, limiting evaluation for intracranial hypertension, cerebral infarcts, brain abscess/empyema, cryptococcoma, or hydrocephalus. Seizures and focal neurological deficits could indicate a focal lesion. His clinical improvement was, therefore, reassuring.

This case also illustrates the lack of intensive monitoring in managing amphotericin B nephrotoxicity in a resource‐limited setting. Conventional amphotericin B commonly induces renal potassium wasting, leading to hypokalemia.

In addition, the patient was lost to follow‐up after discharge and was never reviewed by his initial medical team, so assessment of long‐term neurological outcome was limited.

This case underscores the ethical challenge of managing life‐threatening infections in rural settings with a high patient burden and limited diagnostic and imaging capacity. Clinical decisions were guided by available laboratory findings and patient response to therapy, balancing urgency against potential treatment‐related harm.

### 3.9. Learning Points

1. Global health lessons
  ● CM can occur in patients without identifiable immunosuppression.
  ● Coinfection with bacterial meningitis should be considered when clinical deterioration occurs despite antifungal therapy.
  ● Simple diagnostics such as India ink staining and CrAg testing of CSF remain invaluable in low‐resource settings.
2. Hospital‐based takeaways
  ● Repeat lumbar puncture is critical when patients fail to improve within 48–72 h.
  ● Early CSF evaluation and appropriate antimicrobial therapy improve outcomes in complex CNS infections.
  ● Guideline‐based treatment can be successfully adapted even when advanced diagnostics are unavailable.
3. What could have been done differently
  ● Earlier referral or outsourcing for complete CSF analysis by the referring facility.
  ● Earlier consideration of dual pathology during clinical deterioration.
  ● Earlier empiric antibacterial therapy considering the patient’s acute presentation.
  ● Therapeutic lumbar punctures done for intracranial pressure control.
  ● 40 mEq/day of potassium chloride administered daily to decrease the incidence of amphotericin‐related hypokalemia.
  ● Also, a liposomal formulation of amphotericin considered to lower the risk of overall nephrotoxicity.
  ● Neuroimaging done prior to discharge to rule out complications.
  ● Structured postdischarge follow‐up (a phone call or home visit) planned to assess for long‐term outcomes.

## 4. Conclusion

This case is clinically important because it demonstrates the rare coexistence of CM and polymicrobial bacterial meningitis in an apparently immunocompetent adolescent, diagnosed and managed in a resource‐limited setting without access to neuroimaging, fungal speciation, or advanced immunologic testing. This case highlights how diagnostic reasoning, rather than technology, becomes central to survival in such environments.

CM should remain a key differential diagnosis for subacute meningoencephalitis, even in immunocompetent patients. Concurrent bacterial infection may accelerate disease progression and worsen outcomes. High clinical vigilance and timely use of available diagnostic tools are essential to reduce morbidity and mortality, particularly in resource‐limited settings.

**Nomenclature**
CNS: Central nervous system
CSF: Cerebrospinal fluid
CM: Cryptococcal meningitis
HIV: Human immunodeficiency virus
AIDS: Acquired immunodeficiency syndrome
DM: Diabetes mellitus
CD4: Cluster of differentiation 4
ART: Antiretroviral therapy
CBC: Complete blood count
RBS: Random blood sugar
CrAg: Cryptococcal antigen
TB LAM: Tuberculosis lipoarabinomannan
EZN: Ehrlich–Ziehl–Neelsen
AFBs: Acid‐fast bacilli
IgG: Immunoglobulin G
IgM: Immunoglobulin M
Anti‐GM‐CSF: Anti‐granulocyte‐macrophage colony‐stimulating factor
OD: Once daily
BD: Twice daily
TDS: Thrice daily
QID: Four Times daily
KCl: Potassium chloride
CT: Computed tomography
DIC: Disseminated intravascular coagulation
LP: Lumbar puncture
VP Shunt: Ventriculoperitoneal shunt
GCS: Glasgow Coma Scale
ICP: Intracranial pressure

## Author Contributions

Both authors were involved in the clinical care of the patient, conceptualization of the case report, and collection of the clinical details. Dr. Martha Ann Mbonye drafted and completed the revision of the manuscript.

## Funding

The authors declare that they received no external funding.

## Disclosure

This manuscript has no preprints and has not yet been presented at a scientific conference.

## Ethics Statement

Administrative approval to compile this case report was obtained from Kagando Mission Hospital. Written informed consent to participate was obtained from the patient’s legal guardian.

## Consent

Written informed consent to publish this case report and accompanying clinical information was obtained from the patient’s legal guardian. A copy of the consent form is available for review upon request.

## Conflicts of Interest

The authors declare no conflicts of interest.

## Data Availability

All data generated or analyzed are included in this case report.
